# Structural Basis for the Immunomodulatory Function of Cysteine Protease Inhibitor from Human Roundworm *Ascaris lumbricoides*

**DOI:** 10.1371/journal.pone.0096069

**Published:** 2014-04-29

**Authors:** Guoqiang Mei, Jianmei Dong, Zhaotao Li, Sanling Liu, Yunfeng Liu, Mingze Sun, Guiyun Liu, Zhong Su, Jinsong Liu

**Affiliations:** 1 State Key Laboratory of Respiratory Disease, Guangzhou Institutes of Biomedicine and Health, Chinese Academy of Sciences, Guangzhou, China; 2 School of Life Sciences, University of Science and Technology of China, Hefei, China; The Chinese University of Hong Kong, China

## Abstract

Immunosuppression associated with infections of nematode parasites has been documented. Cysteine protease inhibitor (CPI) released by the nematode parasites is identified as one of the major modulators of host immune response. In this report, we demonstrated that the recombinant CPI protein of *Ascaris lumbricoides* (Al-CPI) strongly inhibited the activities of cathepsin L, C, S, and showed weaker effect to cathepsin B. Crystal structure of Al-CPI was determined to 2.1 Å resolution. Two segments of Al-CPI, loop 1 and loop 2, were proposed as the key structure motifs responsible for Al-CPI binding with proteases and its inhibitory activity. Mutations at loop 1 and loop 2 abrogated the protease inhibition activity to various extents. These results provide the molecular insight into the interaction between the nematode parasite and its host and will facilitate the development of anthelmintic agents or design of anti-autoimmune disease drugs.

## Introduction

Nematode parasite infections that are highly prevalent in many parts of the world cause significant health problems [1]. Infections of these parasites are often characterized by a chronic and asymptomatic course due to the immunosuppression induced by the parasites in the hosts [2]. The nematode-induced immunosuppression is also manifested as protection from autoimmune and allergic diseases [3], [4]. The human gastrointestinal nematode *Ascaris lumbricoides* infects as many as 1.5 billion people globally and causes malnutrition, retarded growth, reduced physical fitness and reduced work capacity in infected individuals. Similar to other nematode parasite, *A. lumbricoides* infection has been shown to modulate hosts' immune responses to infections with unrelated pathogens, reduce incidence of asthma, and lessen skin-test reactivity to allergens and house dust mites [3]–[6]. A recent study showed that *A. lumbricoides* pseudocoelomic fluid modulates dendritic cell phenotype and its function [7]. Therefore, the parasitic nematode extracts were proposed as potential therapeutic agents for treatment of autoimmune disorders and allergic diseases [8], [9].

Cysteine protease inhibitors (CPI; cystatins) from nematodes have been found to play major roles in modulating host immunity [10]–[12]. Cystatins are a group of cysteine protease inhibitors that bind reversibly to cysteine proteases and regulate their proteolytic activities. The predominant target proteases for cystatin are C1 (papain-like cysteine peptidase) and C13 family (legumain). Parasite CPIs can modulate the protease-dependent functions of host immune cells. A number of cathepsins (a mammalian version of the C1 family cysteine proteases) have been identified as important proteases in mediating immune responses, such as proteolytic degradation of the invariant chain that regulates MHC-II molecule intracellular trafficking, antigen processing and cleavage of intracellular domain of Toll-like receptor (TLR)-9 [13]–[15]. Inhibition of these cysteine proteases may suppress the activation of dendritic cells and interfere with the formation of the MHC-II-antigen peptide complex, resulting in impaired ability of the antigen-presenting cell to activate CD4^+^ T cells and immune responses.

The members of cystatin superfamily are categorized into three types based on their amino acid sequences and the position of the disulfide bond(s). Type 1 cystatin (stefins) contains an 11 kD single domain without signal peptide and disulfide bond. Type 2 cystatin, with a molecular weight of ∼13 kD, is a single domain protein secreted into the extracellular region and has two conserved intra-molecular disulfide bonds. Type 3 cystatin (kininogen) comprises three type 2-like domains [16]–[19]. Since the first elucidation of the structure of type 2 chicken egg white (CEW) cystatin by Wolfram Bode [20], many other cystatins structures have been determined by X-ray crystallography or NMR, including human cystatin A-D, F [21]–[26], and cystatins from protozoan parasite and soft tick [27], [28]. Structurally, a typical cystatin fold contains a five-stranded anti-parallel β-sheet wrapped around an α-helix.

Although CPIs from many nematode species have been studied extensively for their roles in the induction of immunosuppression, the structural features of this group of immunomodulatory proteins remain largely unknown. In this study, we found that the recombinant Al-CPI strongly inhibited the proteolytic activity of cathepsins. We then analyzed the structure of *A. lumbricoides* CPI (Al-CPI). We identified the critical segments of Al-CPI molecule that could be involved in the interaction between Al-CPI and its target proteases. Mutagenesis study further confirmed that these segments were responsible for the inhibition of cysteine protease activities.

## Materials and Methods

### Ethics statement

Adult *A. lumbricoides* were collected from patients in a village in Yunnan province, China, after written informed consent. The study protocols were approved by the Institutional Human Study Ethics Committee of Guangzhou Institutes of Biomedicine and Health, Chinese Academy of Sciences.

### Molecular cloning, expression and purification of Al-CPI

To clone the Al-CPI cDNA, total RNA was isolated from adult worms and double-stranded cDNA was obtained by RT-PCR using random primers and a reverse transcription system (Promega, Madison, WI). A fragment of the gene encoding Al-CPI was amplified by PCR from the cDNA with the primers 5′-CCGGAATTCGAAAACCTGTATTTTCAGGGCCAAGTAGGAGTTCCTGGTGGTTTC-3′and 5′-ACGCGTCGACTTATGCAGATTTGCATTCTTTGATG-3′. The sense and antisense primers were designed based on sequences conserved in cystatins previously described for *Nippostrongylus brasiliensis, Onchocerca volvulus, Brugia malayi, Haemonchus contortus* and *Caenorhabditis elegans* in GenBank and *Heligmosomoides polygyrus* [28]. Primers for 3′ and 5′ RACE were synthesized and full-length Al-CPI cDNA was obtained by standard RACE protocols. The full length Al-CPI gene (GenBank accession no. HQ404231) was constructed into pET32a vector and then transformed into Origami (Novagen, Madison, WI). Cells were grown in Luria-Bertani (LB) medium containing 100 mg/ml kanamycin and ampicillin to A_600_ = 0.6–0.8 at 37°C for ∼3 h. The cells were then divided into two parts and cultured at 20°C for 20 h, after being induced with 1 mM isopropyl β-D-1-thiogalactopyranoside (IPTG). Cells were harvested with 6000 rpm centrifugation for 15 min and frozen at −80°C. Al-CPI protein was purified using nickel affinity column with washing buffer A (200 mM NaCl, 20 mM Tris pH 7.5) and then elution buffer B (200 mM NaCl, 250 mM Imidazole, 20 mM Tris pH 7.5), followed by TEV protease digestion in dialysis buffer (100 mM NaCl, 20 mM Tris, pH 7.5). Al-CPI was further purified by size-exclusion chromatography using HiLoad™ 16/60 Superdex™ 75 column (GE Healthcare, Uppsala, Sweden), with a peak elution volume of 83 ml corresponding to the monomeric form of Al-CPI. The expression, purification and crystallization of the recombinant Al-CPI protein were described in detail in our previous report [29]. PCR-based mutagenesis strategy was used to generate Al-CPI mutants and the specific residues predicted to be critical for binding to proteases were replaced. Al-CPI mutants were constructed in pET32a vector and transformed into Origami. The mutant proteins were expressed and purified similarly as the wild type Al-CPI.

### Measurement of cathepsin inhibition activity of Al-CPI

Inhibitory activity of the recombinant Al-CPI and the mutant proteins was determined by protease-activity assays using specific fluorogenic substrates [30]. Cathepsin B and C were purchased from Sigma–Aldrich (St Louis, MO) and cathepsin L and S were obtained from Calbiochem (La Jolla, CA) and Enzo (New York, NY), respectively. The fluorogenic substrates for cathepsin B (Z-Arg-Arg-AMC) and cathepsin C (Gly-Phe β-naphthylamide) were obtained from Sigma-Aldrich and Santa Cruz Biotechnology (Santa Cruz, CA), respectively. Substrates for cathepsin L (Z-Phe-Arg-AMC) and cathepsin S (Z-Val-Val-Arg-AMC) were from Calbiochem and Enzo Life Sciences (Plymouth, PA), respectively. To measure the inhibition activity of Al-CPI, the cathepsins were incubated with the substrates in the absence or presence of serially diluted Al-CPI in appropriate buffer for 15 min. The reaction was stopped with stopping buffer. The amount of product was measured fluorometrically with excitation at 360 nm and emission at 460 nm. The inhibitory activity of Al-CPI was expressed as a percentage of the total activity detected in reactions without Al-CPI. The half maximal inhibitory concentration (IC_50_) of Al-CPI and its mutants based on initial reaction velocities were determined by nonlinear regression analysis.

### Crystallization and X-ray data collection

The monomeric form of Al-CPI eluted from the size-exclusion column was used for crystallization screening. Protein concentration was determined using Bio-Rad Protein assay kit (Bio-Rad, Hercules, CA). Crystals were obtained from 0.2 M sodium acetate trihydrate, 0.1 M sodium cacodylate trihydrate pH 6.5, and 30% w/v polyethylene glycol 8,000 (from Crystal Screen HT). Crystals were grown by the sitting-drop vapor diffusion method and micro-seeding. For data collection, crystals were soaked in the reservoir liquid added with 20% glycerol as the cryo-protectant. X-ray diffraction data were collected to the resolution of 2.1 Å with an in-house Oxford Diffraction Gemini R Ultra system (Oxford, England) and the beam line 17U of the Shanghai Synchrotron Radiation Facility. The diffraction images were indexed and integrated by Mosflm [31] and scaled using SCALA from CCP4 [32]. The crystals belonged to the space group P1. Unit cell parameters are shown in Table 1.

### Structure determination and refinement

Al-CPI structure was determined by molecular replacement using the program MOLREP [33] with chicken egg white cystatin (PDB code: 1CEW) as the search model [20]. The structure was refined by Refmac5 from CCP4 package and rebuilt with Coot [32], [34], [35]. R/Rfree values of the final models are 0.202/0.256. The detailed refinement statistics are shown in Table 1. All structural figures were prepared by PyMOL [36]. Atomic coordinates and structure factors of Al-CPI have been deposited in the Protein Data Bank (PDB) with the accession code 4IT7.

**Table 1:** Data collection and refinement statistics.

	Al-CPI
**Date Collection**	
Wavelength (Å)	1.5405
Temperature (K)	100
R_merge_ a)	0.092 (0.659)
Space Group	P1
Unit cell parameters	
a, b, c (Å)	43.53, 44.50, 45.58
α, β, γ (°)	90.00, 89.99, 90.01
Solvent content (v/v)	27.59%
Unique reflections	19288 (2836)
Completeness	98.8 (99.9)
Multiplicity	3.0 (2.3)
I/σ(I)	9.2 (1.4)
**Refinement**	
Resolution (Å)	18.01–2.10 (2.21–2.10)
No. atoms	
Protein	3220
Water	126
Rb)/R_free_ c)	0.202/0.256
No. reflections	18305
R.m.s. deviationsd)	
Bond lengths (Å)	0.014
Bond angles (°)	1.795
**Ramachandran plot** e)	
Most favorable	91.9%
Additional allowed	8.1%

The values in parentheses refer to statistics in the highest bin.

a) R_merge_ = ∑_hkl_∑_i_|I_i_(hkl)- <I(hkl)>| / ∑_hkl_∑_i_I_i_(hkl), where I_i_(hkl) is the intensity of an observation and <I(hkl)> is the mean value for its unique reflection; summations are over all reflections.

b) R-factor  = ∑_h_|Fo(h)-Fc(h)|/∑_h_Fo(h), where Fo and Fc are the observed and calculated structure-factor amplitudes, respectively.

c) R_free_ was calculated with 5% of the data excluded from the refinement.

d) Root-mean square-deviation from ideal values.

e) Categories were defined by MolProbity.

### Molecular docking analysis

Models for the mutants of Al-CPI were generated using the Design Protein tool in Discover Studio 3.11 (Accelrys Inc., San Diego, CA). Five models of Al-CPI mutants were built and optimized. Docking of wild type and mutated Al-CPI to various cathepsins was done using ZDOCK from Discovery Studio 3.11. Al-CPIs (wild type and mutant forms) and cathepsins were treated as ligands and receptors, respectively. PDB codes for the cathepsins are: 2IPP (cathepsin B), 3PDF (cathepsin C), 3HWN (cathepsin L), 2FQ9 (cathepsin S). In each of the docking calculations, two thousand poses were generated and the poses with a ZDOCK score higher than 12 were refined in RDOCK. The best refined models were chosen for further analysis.

### Statistical analyses

Statistical analyses were performed with GraphPad Prism 5 software (GraphPad Software Inc., La Jolla, CA). Significance of the differences between groups was analyzed using the Student's *t* test. Individual data and mean ± S.D. of the group are presented. A *p* value<0.05 was considered significant.

## Results

### Molecular cloning and biological activity of recombinant Al-CPI

**Figure 1:**
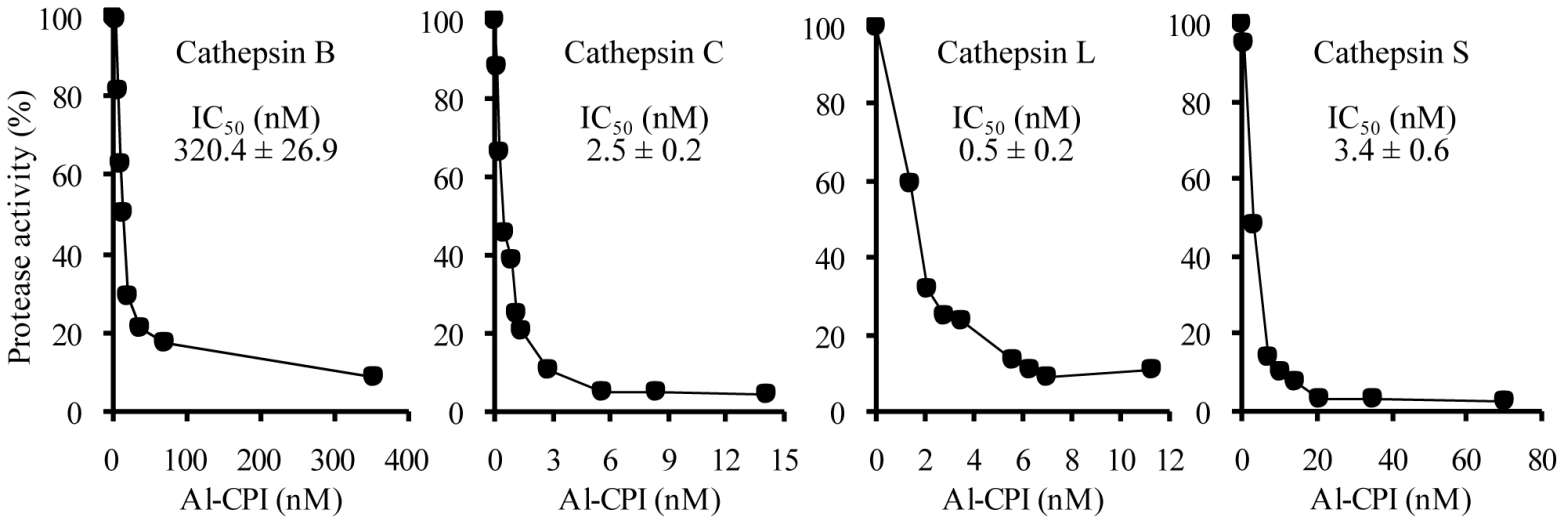
Inhibition activities against cathepsin B, C, L and S by recombinant cysteine protease inhibitor *Ascaris lumbricoides* (Al-CPI). Cathepsins were incubated with the fluorogenic substrates in the absence or presence of serially diluted Al-CPI in appropriate buffer for 15 min. and reaction was stopped with stopping buffer. The amount of product was measured fluorometrically with excitation at 360 nm and emission at 460 nm. The inhibitory activity of Al-CPI was expressed as a percentage of the total activity detected in reactions without Al-CPI. The half maximal inhibitory concentration (IC_50_) values of Al-CPI based on initial reaction velocities were determined by nonlinear regression analysis and are shown on each plot. Data shown are from one of three experiments.

The cDNA library of *A. lumbricoides* was screened by RT-PCR using the primers for consensus sequences of cystatins reported in other nematode parasites, and a fragment of the CPI gene was obtained. The full length CPI gene from *A. lumbricoides* was obtained by RACE technique. The complete cDNA of Al-CPI contains an open reading frame of 399 bp coding for 132 amino acid residues. The biological activity of the recombinant Al-CPI protein was determined by testing its ability to inhibit the proteolytic activity of cathepsin B, C, L and S. The recombinant Al-CPI exhibited various levels of inhibitory activity to the four cathepsins in a dose-dependent manner (Fig. 1). Al-CPI showed strong inhibition to cathepsin L, while intermediate inhibition to cathepsin C, S and weak inhibition to cathepsin B were observed (Fig. 1).

### Structural feature of Al-CPI

**Figure 2:**
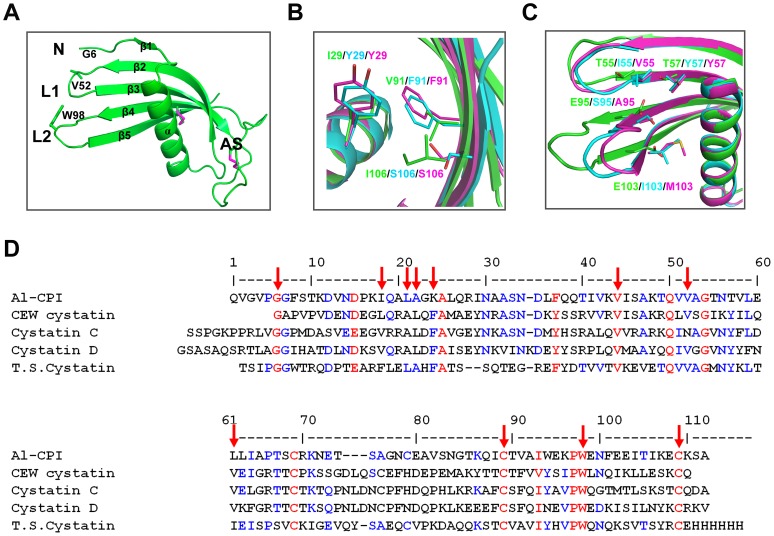
Structure of Al-CPI and structural comparison of Al-CPI with CEW cystatin and human cystatin C. (A) Monomer structure of Al-CPI. (B) Structural comparison of Al-CPI (green), CEW cystatin (cyan), and cystatin C (magenta) around the α-helix core. (C) Structural comparison of Al-CPI (green), CEW cystatin (cyan) and cystatin C (magenta) around the active site segment. (D) Sequence alignment of five type-2 cystatins from *A. lumbricoides*, chicken egg white (CEW) and soft tick salivary gland (T.S. cystatin) and human cystatin C and D. The amino acid residues partially conserved among the five cystatins are highlighted in blue, and fully conserved are highlighted in red. The amino acid residues indexed with red arrow were selected for distance measurement as shown in Table 2.

To further understand the molecular mechanism of the interaction between Al-CPI and its target proteases, crystal structure of Al-CPI protein was obtained. The monomeric form of Al-CPI crystallized in the space group P1 (Table 1). There are four copies of Al-CPI monomer in the asymmetric unit. The Al-CPI monomer structure shows a conventional type-2 cystatin fold. It has a five-stranded anti-parallel β-sheet that wraps around the central α-helix. From the N-terminus to the C-terminus, Al-CPI contains: N-terminal fragment (N), short β-strand 1 (β1, residue 9-11), α-helix (17–32), β2 (31–50), loop1 (L1, 51–54), β3 (55–64), appending structure (AS, 65–86), β4 (87–96), loop2 (L2, 97–101), and β5 (102–112). Al-CPI also has two conserved intra-molecular disulfide bridges between C68 and C78 and between C89 and C109 (Fig. 2A). In the final model, the N-terminal five residues were invisible from the electron density map and were not modelled. Similar to crystal structures of cystatin and cathepsin complex reported previously by others [37], the N-terminal fragment (G6-G7), loop 1 (V51-T54) and loop 2 (P97-F101) of Al-CPI form a wedge segment that is likely to insert into the activity pocket of papain-like cysteine proteases in such a way that Al-CPI can inhibit the protease activity.

Only five unique types-2 cystatin structures were found in PDB to this date. They are from different species: CEW cystatin from chicken (*Gullus gallus*), cystatin C, D and F from human (*Homo sapiens*), and salivary cystatin from soft tick (*Ornithodoros moubata*). Among these five structures, CEW cystatin has the highest sequence identity (34%) with Al-CPI and cystatin C shows the highest structure similarity with Al-CPI with a Z-score of 16.0 from Dali server [38]. Most of these cystatin structures, including a V57N mutated form of cystatin C, are monomer. One exception is cystatin F that was glycosylated and formed a dimer in the structure. To compare the structures of these similar cystatins, multiple sequence alignment was performed with Multalin. The distances between the α-helix and other parts of cystatins were then measured using the Cα of conserved amino acid residues (marked by red arrow in Fig. 2D). The distance between the α-helix and the β-sheet was much shorter in Al-CPI and tick salivary cystatin, compared with other cystatins. The distance between the α-helix and the active site segment (N, L1 and L2) was longer in Al-CPI and salivary cystatin than the distance in other cystatins (Table 2). As tick salivary cystatin is very similar to Al-CPI in this local region, for clarity we only superimposed the structures of Al-CPI with CEW cystatin and cystatin C. As shown in Fig. 2B and C,the α-helix core packs much tighter against the β-sheet in Al-CPI than in CEW cystatin and cystatin C; the active site segment (N, L1 and L2) moves away from the α-helix and becomes more open in Al-CPI.

**Table 2:** Comparison of Al-CPI with other monomeric type-2 cystatinsa).

		CEW cystatin	Cystatin C	Cystatin D	Al-CPI	T.S. Cystatin
Distance between α-helix and β-sheet (Å)	β2	L22-V44 9.0	L22-V44 8.4	L22-V44 9.1	A22-V44 7.2	A22-V44 7.4
	β3	L22-V61 11.4	L22-V61 8.6	L22-V61 9.3	A22-L61 7.4	A22-I61 7.3
	β4	L22-C89 13.6	L22-C89 11.1	L22-C89 12.0	A22-C89 10.6	A22-C89 10.1
	β5	L22-C109 14.8	L22-C109 14.1	L22-109 15.1	A22-C109 14.1	A22-C109 13.3
Distance between α-helix and active site (Å)	N	L18-G6 18.5	V18-G6 14.6	V18-G6 17.3	I18-G6 19.7	F18-G6 20.3
	L1	A21-V52 16.0	A21-N52 16.3	A21-V52 18.2	L21-V52 19.1	L21-V52 19.1
	L2	F24-W98 18.2	F24-W98 16.8	F24-W98 16.8	K24-W98 20.3	F24-W98 18.3

a) The Cα of conserved amino acid residues were chosen for all the distance measurements.

A detailed analysis of the residues involved in the intra-molecular packing interface reveals that Al-CPI has some unique sequence features not observed in other cystatins. Al-CPI has an isoleucine (I29) in the middle of the α-helix, while the amino acid at that position in other cystatins is a tyrosine (Fig. 2B). Directly across that position, there is a valine (V91) in Al-CPI while in other cystatins there is instead a phenylalanine. The bulky aromatic residues will push the α-helix away from the β-sheet. A third position is I106 for Al-CPI, while it is a serine in other cystatins. A hydrophobic residue (isoleucine) will help the α-helix pack closer to the β-sheet (to become a hydrophobic core) than a hydrophilic residue such as serine can do. These sequence differences also exist in other cystatins (data not shown). Interestingly, in the interface between the active site segment (N, L1 and L2) and the α-helix, Al-CPI contains mostly polar residues while other cystatins contain mostly hydrophobic residues (Fig. 2C). Therefore, compared to the active site segment of Al-CPI, the active site segment of other cystatins packs closer to the α-helix.

### Interaction between Al-CPI and cathepsins

**Figure 3:**
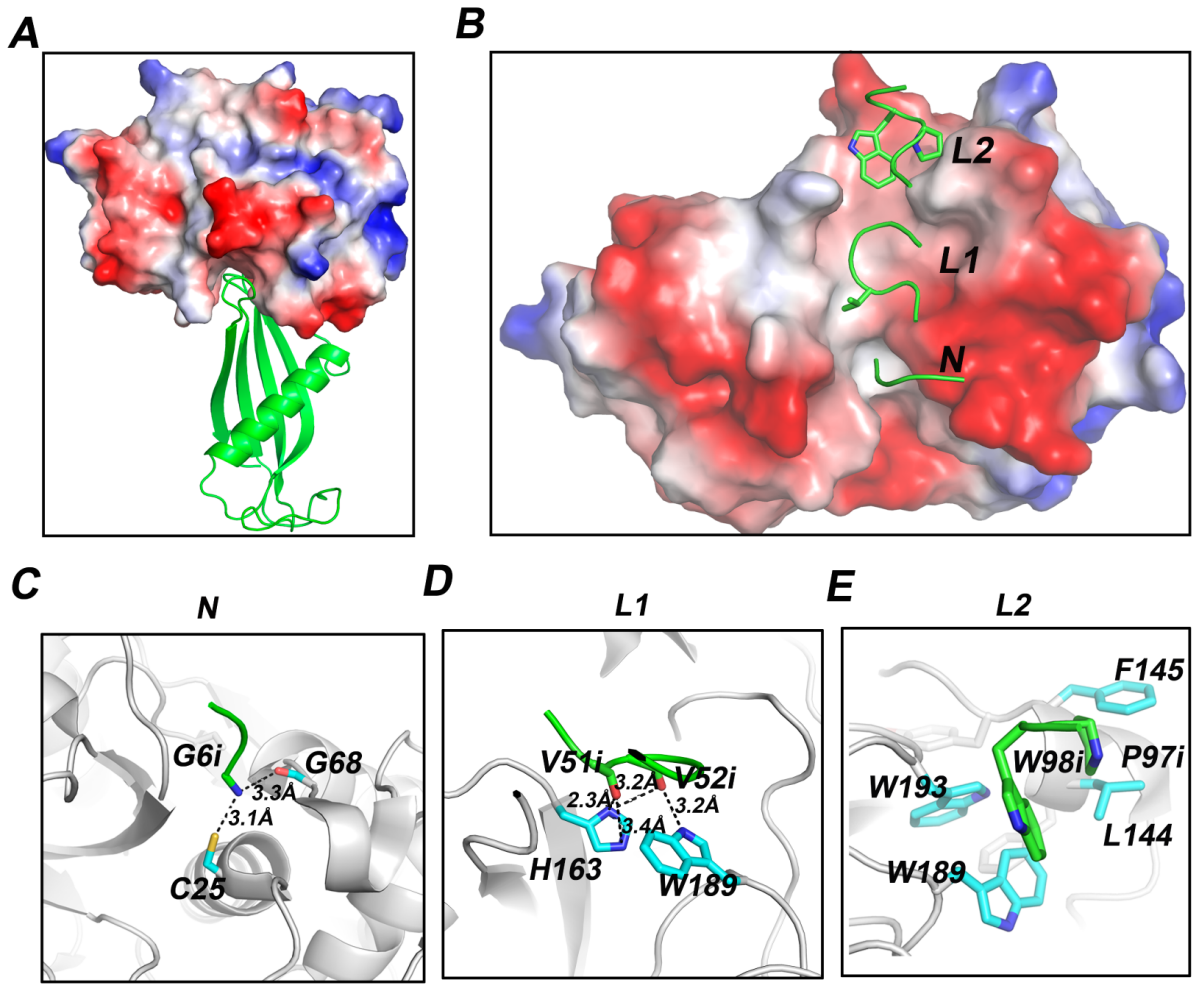
Docking analysis of Al-CPI with cathepsin L. (A) Docking between Al-CPI and cathepsin L. (B) 90° rotation around the x-axis from the view of (A). (C–E) The detailed analysis of interactions between cathepsin L and Al-CPI N-terminal (C), loop 1 (D) and loop 2 (E). Inhibitor (Al-CPI) residues are indicated with an “i” after the sequence number to distinguish them from those of the enzyme (cathepsin).

The docking analysis revealed that the interaction between Al-CPI and cathepsin L mainly involved a hydrophobic groove in cathepsin L (Fig. 3A and B). H163 and C25, being the key residues responsible for the cathepsin activity, could form hydrogen bonds with the main chains of G6, V51 and V52 in Al-CPI (Fig. 3C and D). Additionally, P97 and W98 from Al-CPI would pack against L114, F145, W189 and W193 in cathepsin L (Fig. 3E). These interactions revealed by the docking analysis suggest that three regions of Al-CPI (G6 of N-terminal fragment, V51 and V52 of loop 1, and P97 and W98 of loop 2) may be important for the Al-CPI binding with the cathepsins to exert its inhibitory effect.

**Figure 4:**
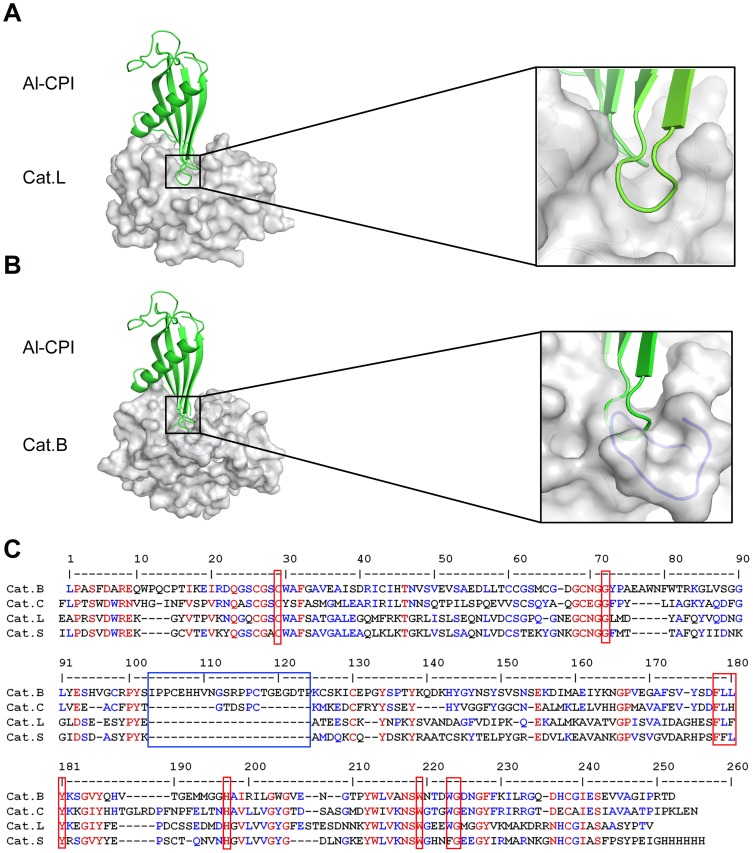
Structural basis of differential inhibition activity of Al-CPI to cathepsin L (Cat. L) and cathepsin B (Cat. B). (A) Molecular docking was performed for Al-CPI and Cat. L. (B) Docking of AL-CPI against Cat. B suggested the insert segment of Cat. B would crash with the L2 of Al-CPI. (C) The sequence alignment of four cathepsins showing the unique insert segment of Cat. B.

The enzymatic experiment results showed the strongest inhibitory potency of Al-CPI to cathepsin L but the weakest to cathepsin B (Fig. 1). To further understand the molecular basis of this difference, we performed docking calculations for four cathepsins. Al-CPI exhibits the highest binding affinity with cathepsin L and the lowest affinity with cathepsin B among the four cathepsins tested (Fig. 4A and B; Table 3). The docking calculations support and provide a possible explanation to the experimental inhibition data. Sequence alignment shows that the active site is well conserved across different cathepsins (Fig. 4C, shown in red box). However, cathepsin B has a unique insert sequence (105–124) (Fig. 4C, shown in blue box). Docking analysis results indicate that the insert segment of cathepsin B would crash with L2 of Al-CPI, resulting in reduced binding affinity and inhibition activity (Fig. 4B).

**Table 3:** The docking energy between Al-CPI and various enzymes.

	Inhibitor	Enzyme	E-RDocka) (kcal/mol)
	Al-CPI	Cathepsin B	−3.26
Wild-type Al-CPI	Al-CPI	Cathepsin C	-13.81
	Al-CPI	Cathepsin L	−18.10
	Al-CPI	Cathepsin S	−7.81
	G6E (Mutant 1)	Cathepsin L	−11.65
	V52G (Mutant 2)	Cathepsin L	−16.07
Mutant Al-CPI	Q50E V52G (Mutant 3)	Cathepsin L	−12.36
	P97G W98G (Mutant 4)	Cathepsin L	−3.7
	G6E Q50E V52G P97G W98G (Mutant 5)	Cathepsin L	−1.14

a) E_RDock, the RDOCK score is defined as: E_elec2+beta×E_sol. E_elec2: the electrostatic energy of the protein complex after the first and second CHARMm minimizations. E_sol: the desolvation energy of the protein complex calculated by the ACE method.

### Protease inhibition activity of Al-CPI variants

**Figure 5:**
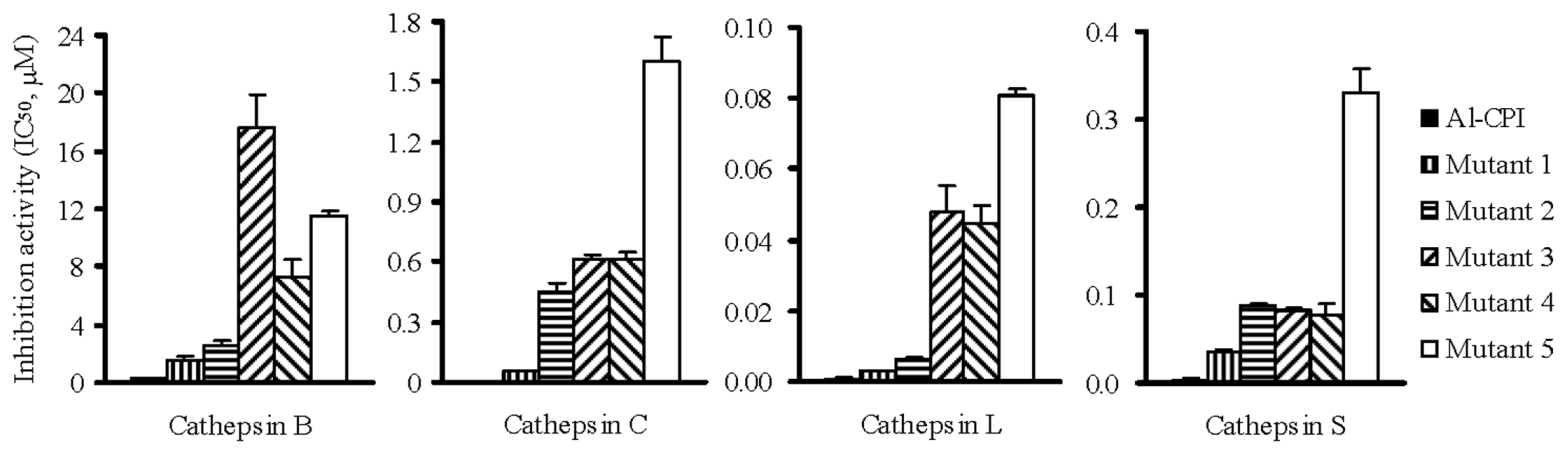
Protease inhibition of wild-type Al-CPI and the mutants. IC_50_ values of wild type Al-CPI and five mutated forms to cathepsin B, C, L and S were shown. The inhibition activities of Al-CPI and its mutants were analyzed as described in Materials and.

The structural analysis results presented above suggest that the G6 of N-terminal fragment, loop 1 and loop 2 of Al-CPI are critical regions for Al-CPI to bind to the proteases. To verify the importance of these regions in Al-CPI function, Al-CPI mutants were generated and tested for their protease inhibition activities. Dramatic changes in the protease inhibition were observed in Q50E+V52G (mutant 3) and P97G+W98G (mutant 4) double mutations and combined mutations (mutant 5). These mutants exhibited significantly reduced inhibition activities to cathepthins (increased IC_50_ values) (Fig. 5). Mutations at the critical binding sites also resulted in significant changes in the calculated binding affinity between Al-CPI variants and cathepsin L (Table 3). Compared with the wild-type Al-CPI, V52G mutant showed slightly reduced binding affinity to cathepsin L as well as slightly reduced enzymatic inhibition activity to cathepsin C and S. However, mutations of Q50E+V52G (loop 1) resulted in greater reduction in the affinity and protease inhibition activity than the V52G single mutation. From docking calculation, the G6E mutant showed reduced binding affinity to cathepsin L to a greater extent than that shown by the V52G mutant (Table 3). Yet, in enzymatic assay, the inhibitory activity was reduced to a smaller extent in the G6E mutant than in the V52G mutant (Fig. 5). This discrepancy is likely due to some additional effects induced by the changes around the V52 region that were not factored in the docking calculations.

## Discussion

Nematode parasites are known to modulate or suppress the immune responses of host and, consequently, protect against the development of autoimmune and allergic diseases [2], [39], [40]. Although CPIs of nematode parasites have been studied extensively for their immunomodulation function, the knowledge of their detailed structural features is still lacking. Elucidation of the structure and function relationship of parasite CPIs would greatly facilitate the discovery of a new group of drugs for treatment of allergic diseases. To this end, we investigated the structural basis of the immunomodulatory function of CPI from *A. lumbricoides*. We identified the N-terminal segment, the loop 1 and the loop 2 as the key regions for Al-CPI binding with cathepsins. These observations are consistent with those previously reported for other cystatins [20], [21], [37]. However, our results further showed that only the mutations at loop 1 and loop 2 significantly reduced the inhibition activity of Al-CPI. Docking analysis between Al-CPI and four cathepsins demonstrated various binding affinities that were consistent with the inhibition activities detected in the enzymatic analysis. Al-CPI has less inhibitory activity against cathepsin B, compared with C, L and S. Structural and docking analysis suggested that the specificity is mainly due to the insertion of a short segment in cathepsin B, causing the steric hindrance for Al-CPI binding. These results revealed the details of the potential molecular interaction between Al-CPI and the proteases, identified the regions critical for Al-CPI inhibition functions, and provided explanation to the differential inhibition activities of Al-CPI against the four cathepsins studied.

Compared with other type-2 cystatins, we found that the parasite cystatins (both Al-CPI and tick salivary cystatin) had two unique structure features: a tighter hydrophobic core and a more open active site segment. This could be related to their functions during evolution. In a parasite's life cycle, its cystatins not only have to act on its own proteases, but also on proteases in its host. The relatively tighter core may prevent the cystatins from being degraded easily in the hosts. The more open active site segment could render the cystatins more flexible and accessible for binding to the target cysteine protease. This hypothesis should be verified by further experiments.

In conclusion, our results demonstrated that the cysteine protease inhibitor from human gastrointestinal nematode, *A. lumbricoides*, has distinctive effect on different cathepsins. Structural analysis of the recombinant Al-CPI protein identified the key segments involved in the enzymatic function of this parasite-derived molecule. These observations may provide important insight into the molecular mechanism of immunosuppression associated with helminth infections and might be useful for the development of anti-allergic immunomodulatory drugs.

## References

[1] Brooker S (2010) Estimating the global distribution and disease burden of intestinal nematode infections: adding up the numbers—a review. Int J Parasitol 40: 1137–1144. 20430032 10.1016/j.ijpara.2010.04.004 PMC3034165

[2] McSorley HJ, Maizels RM (2012) Helminth infections and host immune regulation. Clin Microbiol Rev 25: 585–608. 23034321 10.1128/CMR.05040-11 PMC3485755

[3] Nacher M, Singhasivanon P, Yimsamran S, Manibunyong W, Thanyavanich N, et al (2002) Intestinal helminth infections are associated with increased incidence of Plasmodium falciparum malaria in Thailand. J Parasitol 88: 55–58. 12053980 10.1645/0022-3395(2002)088[0055:IHIAAW]2.0.CO;2

[4] Flohr C, Tuyen LN, Lewis S, Quinnell R, Minh TT, et al (2006) Poor sanitation and helminth infection protect against skin sensitization in Vietnamese children: A cross-sectional study. J Allergy Clin Immunol 118: 1305–1311. 17157661 10.1016/j.jaci.2006.08.035

[5] Selassie FG, Stevens RH, Cullinan P, Pritchard D, Jones M, et al (2000) Total and specific IgE (house dust mite and intestinal helminths) in asthmatics and controls from Gondar, Ethiopia. Clin Exp Allergy 30: 356–358. 10691893 10.1046/j.1365-2222.2000.00706.x

[6] Nyan OA, Walraven GE, Banya WA, Milligan P, Van Der Sande M, et al (2001) Atopy, intestinal helminth infection and total serum IgE in rural and urban adult Gambian communities. Clin Exp Allergy 31: 1672–1678. 11696042 10.1046/j.1365-2222.2001.00987.x

[7] Dowling DJ, Noone CM, Adams PN, Vukman KV, Molloy SF, et al (2011) Ascaris lumbricoides pseudocoelomic body fluid induces a partially activated dendritic cell phenotype with Th2 promoting ability in vivo. Int J Parasitol 41: 255–261. 20974144 10.1016/j.ijpara.2010.09.007

[8] Johnston MJ, MacDonald JA, McKay DM (2009) Parasitic helminths: a pharmacopeia of anti-inflammatory molecules. Parasitology 136: 125–147. 19079844 10.1017/S0031182008005210

[9] Harnett W, Harnett MM (2010) Helminth-derived immunomodulators: can understanding the worm produce the pill? Nat Rev Immunol 10: 278–284. 20224568 10.1038/nri2730

[10] Dainichi T, Maekawa Y, Ishii K, Zhang T, Nashed BF, et al (2001) Nippocystatin, a cysteine protease inhibitor from Nippostrongylus brasiliensis, inhibits antigen processing and modulates antigen-specific immune response. Infect Immun 69: 7380–7386. 11705911 10.1128/IAI.69.12.7380-7386.2001 PMC98825

[11] Schonemeyer A, Lucius R, Sonnenburg B, Brattig N, Sabat R, et al (2001) Modulation of human T cell responses and macrophage functions by onchocystatin, a secreted protein of the filarial nematode Onchocerca volvulus. J Immunol 167: 3207–3215. 11544307 10.4049/jimmunol.167.6.3207

[12] Hartmann S, Lucius R (2003) Modulation of host immune responses by nematode cystatins. Int J Parasitol 33: 1291–1302. 13678644 10.1016/s0020-7519(03)00163-2

[13] Riese RJ, Chapman HA (2000) Cathepsins and compartmentalization in antigen presentation. Curr Opin Immunol 12: 107–113. 10679409 10.1016/s0952-7915(99)00058-8

[14] Chapman HA (2006) Endosomal proteases in antigen presentation. Curr Opin Immunol 18: 78–84. 16338127 10.1016/j.coi.2005.11.011

[15] Park B, Brinkmann MM, Spooner E, Lee CC, Kim YM, et al (2008) Proteolytic cleavage in an endolysosomal compartment is required for activation of Toll-like receptor 9. Nat Immunol 9: 1407–1414. 18931679 10.1038/ni.1669 PMC2735466

[16] Turk V, Brzin J, Kotnik M, Lenarcic B, Popovic T, et al (1986) Human cysteine proteinases and their protein inhibitors stefins, cystatins and kininogens. Biomed Biochim Acta 45: 1375–1384. 3495261

[17] Rawlings ND, Barrett AJ (1990) Evolution of proteins of the cystatin superfamily. J Mol Evol 30: 60–71. 2107324 10.1007/BF02102453

[18] Otto HH, Schirmeister T (1997) Cysteine Proteases and Their Inhibitors. Chem Rev 97: 133–172. 11848867 10.1021/cr950025u

[19] Barrett AJ, Fritz H, Grubb A, Isemura S, Jarvinen M, et al (1986) Nomenclature and classification of the proteins homologous with the cysteine-proteinase inhibitor chicken cystatin. Biochem J 236: 312. 3491603 10.1042/bj2360312 PMC1146824

[20] Bode W, Engh R, Musil D, Thiele U, Huber R, et al (1988) The 2.0 A X-ray crystal structure of chicken egg white cystatin and its possible mode of interaction with cysteine proteinases. EMBO J 7: 2593–2599. 3191914 10.1002/j.1460-2075.1988.tb03109.x PMC457133

[21] Stubbs MT, Laber B, Bode W, Huber R, Jerala R, et al (1990) The refined 2.4 A X-ray crystal structure of recombinant human stefin B in complex with the cysteine proteinase papain: a novel type of proteinase inhibitor interaction. EMBO J 9: 1939–1947. 2347312 10.1002/j.1460-2075.1990.tb08321.x PMC551902

[22] Martin JR, Craven CJ, Jerala R, Kroon-Zitko L, Zerovnik E, et al (1995) The three-dimensional solution structure of human stefin A. J Mol Biol. 246: 331–343. 10.1006/jmbi.1994.0088 7869384

[23] Alvarez-Fernandez M, Liang YH, Abrahamson M, Su XD (2005) Crystal structure of human cystatin D, a cysteine peptidase inhibitor with restricted inhibition profile. J Biol Chem 280: 18221–18228. 15728581 10.1074/jbc.M411914200

[24] Janowski R, Kozak M, Abrahamson M, Grubb A, Jaskolski M (2005) 3D domain-swapped human cystatin C with amyloidlike intermolecular beta-sheets. Proteins 61: 570–578. 16170782 10.1002/prot.20633

[25] Schuttelkopf AW, Hamilton G, Watts C, van Aalten DM (2006) Structural basis of reduction-dependent activation of human cystatin F. J Biol Chem. 281: 16570–16575. 10.1074/jbc.M601033200 16601115

[26] Orlikowska M, Jankowska E, Kolodziejczyk R, Jaskolski M, Szymanska A (2011) Hinge-loop mutation can be used to control 3D domain swapping and amyloidogenesis of human cystatin C. J Struct Biol. 173: 406–413. 10.1016/j.jsb.2010.11.009 21074623

[27] Salat J, Paesen GC, Rezacova P, Kotsyfakis M, Kovarova Z, et al (2010) Crystal structure and functional characterization of an immunomodulatory salivary cystatin from the soft tick Ornithodoros moubata. Biochem J 429: 103–112. 20545626 10.1042/BJ20100280 PMC3523712

[28] Hansen G, Heitmann A, Witt T, Li H, Jiang H, et al (2011) Structural basis for the regulation of cysteine-protease activity by a new class of protease inhibitors in Plasmodium. Structure 19: 919–929. 21742259 10.1016/j.str.2011.03.025

[29] Liu S, Dong J, Mei G, Liu G, Xu W, et al (2011) Crystallization and preliminary crystallographic studies of a cysteine protease inhibitor from the human nematode parasite Ascaris lumbricoides. Acta Crystallogr Sect F Struct Biol Cryst Commun 67: 228–230. 10.1107/S1744309110050773 PMC3034614 21301092

[30] Dolenc I, Turk B, Pungercic G, Ritonja A, Turk V (1995) Oligomeric structure and substrate induced inhibition of human cathepsin C. J Biol Chem. 270: 21626–21631. 10.1074/jbc.270.37.21626 7665576

[31] Leslie AGW, Powell HR (2007) Processing diffraction data with MOSFLM. Evolving Methods for Macromolecular Crystallography 245: 41–51.

[32] Collaborative Computational Project N (1994) The CCP4 suite: programs for protein crystallography. Acta Crystallogr D Biol Crystallogr 50: 760–763. 15299374 10.1107/S0907444994003112

[33] Teplyakov AVaA (1997) MOLREP: an Automated Program for Molecular Replacement. J Appl Cryst 30: , 1022–1025

[34] Murshudov GN, Skubak P, Lebedev AA, Pannu NS, Steiner RA, et al (2011) REFMAC5 for the refinement of macromolecular crystal structures. Acta Crystallogr D Biol Crystallogr 67: 355–367. 21460454 10.1107/S0907444911001314 PMC3069751

[35] Emsley P, Cowtan K (2004) Coot: model-building tools for molecular graphics. Acta Crystallogr D Biol Crystallogr 60: 2126–2132. 15572765 10.1107/S0907444904019158

[36] DeLano W (2002) The PyMOL Molecular Graphics System.

[37] Jenko S, Dolenc I, Guncar G, Dobersek A, Podobnik M, et al (2003) Crystal structure of Stefin A in complex with cathepsin H: N-terminal residues of inhibitors can adapt to the active sites of endo- and exopeptidases. J Mol Biol 326: 875–885. 12581647 10.1016/s0022-2836(02)01432-8

[38] Holm L, Rosenstrom P (2010) Dali server: conservation mapping in 3D. Nucleic Acids Res 38: W545–549. 20457744 10.1093/nar/gkq366 PMC2896194

[39] Kabeerdoss J, Pugazhendhi S, Subramanian V, Binder HJ, Ramakrishna BS (2011) Exposure to hookworms in patients with Crohn's disease: a case-control study. Aliment Pharmacol Ther 34: 923–930. 21848628 10.1111/j.1365-2036.2011.04824.x

[40] Santiago HC, Bennuru S, Boyd A, Eberhard M, Nutman TB (2011) Structural and immunologic cross-reactivity among filarial and mite tropomyosin: implications for the hygiene hypothesis. J Allergy Clin Immunol 127: 479–486. 21185070 10.1016/j.jaci.2010.11.007 PMC3075728

